# Physical Activity in Arrhythmogenic Cardiomyopathy: From Trigger to Therapeutic Dilemma

**DOI:** 10.31083/RCM52871

**Published:** 2026-08-18

**Authors:** Elham Kayvanpour, Benjamin Meder, Farbod Sedaghat-Hamedani

**Affiliations:** ^1^Rehabilitation Clinic Heidelberg-Koenigstuhl, 69117 Heidelberg, Germany; ^2^Institut für Cardiomyopathien Heidelberg, University of Heidelberg, 69120 Heidelberg, Germany; ^3^German Centre for Cardiovascular Research (DZHK), Partner Site Heidelberg/Mannheim, 69120 Heidelberg, Germany

**Keywords:** arrhythmogenic right ventricular dysplasia, endurance training, cardiac arrhythmias, desmoplakin, exercise therapy, ventricular remodelling

## Abstract

Arrhythmogenic cardiomyopathy (ACM) is highly sensitive to environmental influences, with physical exercise emerging as one of the most important determinants of disease expression. Growing evidence indicates that high-intensity and endurance exercise accelerate structural remodelling, promote inflammatory myocardial injury, and increase the incidence of malignant ventricular arrhythmias in genetically predisposed individuals. These effects extend beyond acute exertion, as cumulative exercise exposure contributes to the development of a persistent arrhythmogenic substrate that may sustain risk even after cessation of competitive sport. In contrast, available data suggest that low-intensity physical activity does not confer the same degree of arrhythmic burden and may support overall cardiovascular and functional health when carefully prescribed. This review integrates mechanistic insights, clinical cohort data, and current guideline recommendations to provide a balanced framework for exercise counselling in ACM. Rather than advocating complete physical inactivity, this approach differentiates high-risk endurance or competitive exercise from carefully selected low-intensity recreational activity and emphasizes individualized, shared decision-making. Thus, understanding the interaction among genetic substrate, exercise intensity, and systemic stressors is essential for optimizing personalized activity recommendations and improving long-term outcomes in this vulnerable population.

## 1. Introduction

Arrhythmogenic cardiomyopathy (ACM) is an inherited myocardial disorder characterized by ventricular arrhythmias, progressive structural remodelling, and an increased risk of sudden cardiac death (SCD) [1]. Initially described as a predominantly right ventricular disease, ACM is now recognized as a heterogeneous spectrum that includes right-dominant, left-dominant, and biventricular phenotypes. Advances in molecular genetics have identified pathogenic variants in desmosomal and non-desmosomal genes as key determinants of disease susceptibility [2,3,4]. However, the marked variability in clinical presentation and progression indicates that genetic background alone does not fully determine phenotype, which explains the role of environmental modifiers [5].

Physical exercise is one of the most clinically significant factors influencing disease expression in ACM. In contrast to most cardiovascular disorders, where regular activity is strongly encouraged, high-intensity and endurance exercise in ACM has been consistently associated with earlier phenotypic expression, greater structural remodelling, and increased arrhythmic risk [6]. Experimental and observational evidence suggests that repetitive mechanical stress imposed on a genetically vulnerable myocardium accelerates disease progression rather than acting solely as an acute arrhythmic trigger [5,7,8]. Importantly, exercise counselling in ACM should not be interpreted as a universal recommendation for complete physical inactivity. Contemporary sports cardiology practice has moved toward individualized risk assessment and shared decision-making, acknowledging that the risk associated with physical activity varies according to disease stage, genotype, ventricular involvement, arrhythmic history, imaging findings, exercise intensity, and the type of sport performed [9]. While sustained high-intensity and competitive endurance exercise remain strongly discouraged in many patients with overt ACM or significant arrhythmic burden, carefully selected low-intensity recreational activity may be appropriate and may help preserve functional capacity, cardiometabolic health, and psychosocial well-being [9,10,11,12]. Therefore, the central clinical challenge is not whether all exercise should be prohibited, but how to define a safe and individualized activity range that minimizes arrhythmic and structural risk while avoiding the adverse consequences of unnecessary inactivity. This review examines the mechanistic basis, clinical evidence, and current guideline perspectives regarding physical activity in ACM, with the aim of providing a balanced and risk-adapted framework for individualized exercise counselling and shared decision-making.

## 2. Clinical Associations Between ACM and Physical Activity

Clinical evidence consistently supports an association between physical activity, particularly sustained high-intensity and endurance exercise, and adverse disease expression in ACM. Across disease-based and family-based cohorts, exercise has been identified as an important environmental modifier that may influence age at disease onset, ventricular remodelling, arrhythmic burden, and clinical outcomes in susceptible individuals carrying pathogenic or likely pathogenic ACM-associated variants [5,13,14]. These observations provide the clinical basis for considering exercise exposure as a relevant component of risk assessment in ACM. Importantly, these associations do not imply that all forms of physical activity carry the same risk. Available evidence suggests that exercise-related risk is shaped by cumulative exposure, intensity, disease stage, genotype, ventricular involvement, arrhythmic history, imaging findings, and the type of sport performed [7,9,13]. Therefore, the clinical challenge is not to recommend complete physical inactivity, but to distinguish high-risk competitive or endurance exercise from carefully selected low-intensity recreational activity within an individualized shared decision-making framework.

## 3. Dose–Response Relationship and Endurance Exercise

A consistent and reproducible finding across ACM research is the presence of a dose–response relationship between exercise exposure and adverse clinical outcomes [13]. Multiple observational cohorts have demonstrated that higher cumulative exercise volumes are associated with earlier phenotypic expression, a higher incidence of sustained ventricular tachycardia (VT) and ventricular fibrillation (VF), increased rates of appropriate implantable cardioverter-defibrillator (ICD) therapies, and accelerated progression to ventricular dilation and systolic dysfunction [5,11,13,14,15]. Importantly, these associations remain significant after adjustment for age, sex, and baseline disease severity, supporting cumulative exercise exposure as an independent modifier of disease trajectory rather than merely a lifestyle correlate.

Beyond total exercise volume, intensity has emerged as a critical determinant of risk [7,13]. High-intensity endurance activities, including long-distance running, competitive cycling, swimming, and triathlon training, are associated with disproportionately greater structural and arrhythmic risk compared with low-to-moderate intensity activity, even when overall exercise duration is similar. This association is biologically plausible because sustained high-intensity exercise exposes the heart to prolonged increases in preload, wall stress, and repetitive stretch. In genetically susceptible individuals, these mechanical forces place additional strain on the intercalated disc and may aggravate underlying desmosomal dysfunction [7].

Clinically, this intensity-dependent risk gradient is reflected in the over-representation of endurance athletes among individuals with ACM presenting with exercise-related ventricular arrhythmias or SCD [11,15]. Long-term participation in high-intensity endurance sport appears to accelerate disease penetrance and promote earlier development of an arrhythmogenic substrate through progressive myocardial remodelling, fibrosis, and electrical instability [5]. Consequently, individuals with underlying ACM who engage in sustained high-intensity training may be particularly susceptible to malignant ventricular arrhythmias during exertion and in the early recovery phase, compared with those participating in lower-intensity recreational activity [11,16,17]. Collectively, the available evidence supports a graded model in which cumulative exposure and exercise intensity interact to influence structural progression and arrhythmic vulnerability. These findings have direct implications for risk stratification and exercise counselling in patients with established ACM and in clinically evaluated genotype-positive individuals carrying pathogenic or likely pathogenic ACM-associated variants. However, recommendations for incidentally identified genotype-positive but phenotype-negative individuals from population-based genomic screening require additional caution, because penetrance appears lower and the available evidence on exercise-related disease modification in this setting remains limited. In line with current guideline recommendations, sustained high-intensity and competitive endurance sports should generally be discouraged in patients with overt ACM or significant arrhythmic burden, whereas carefully selected low-intensity recreational activity may be considered after individualized clinical assessment and shared decision-making [9,18].

## 4. Potential Benefits of Low-Intensity Physical Activity

**Fig. 1.**
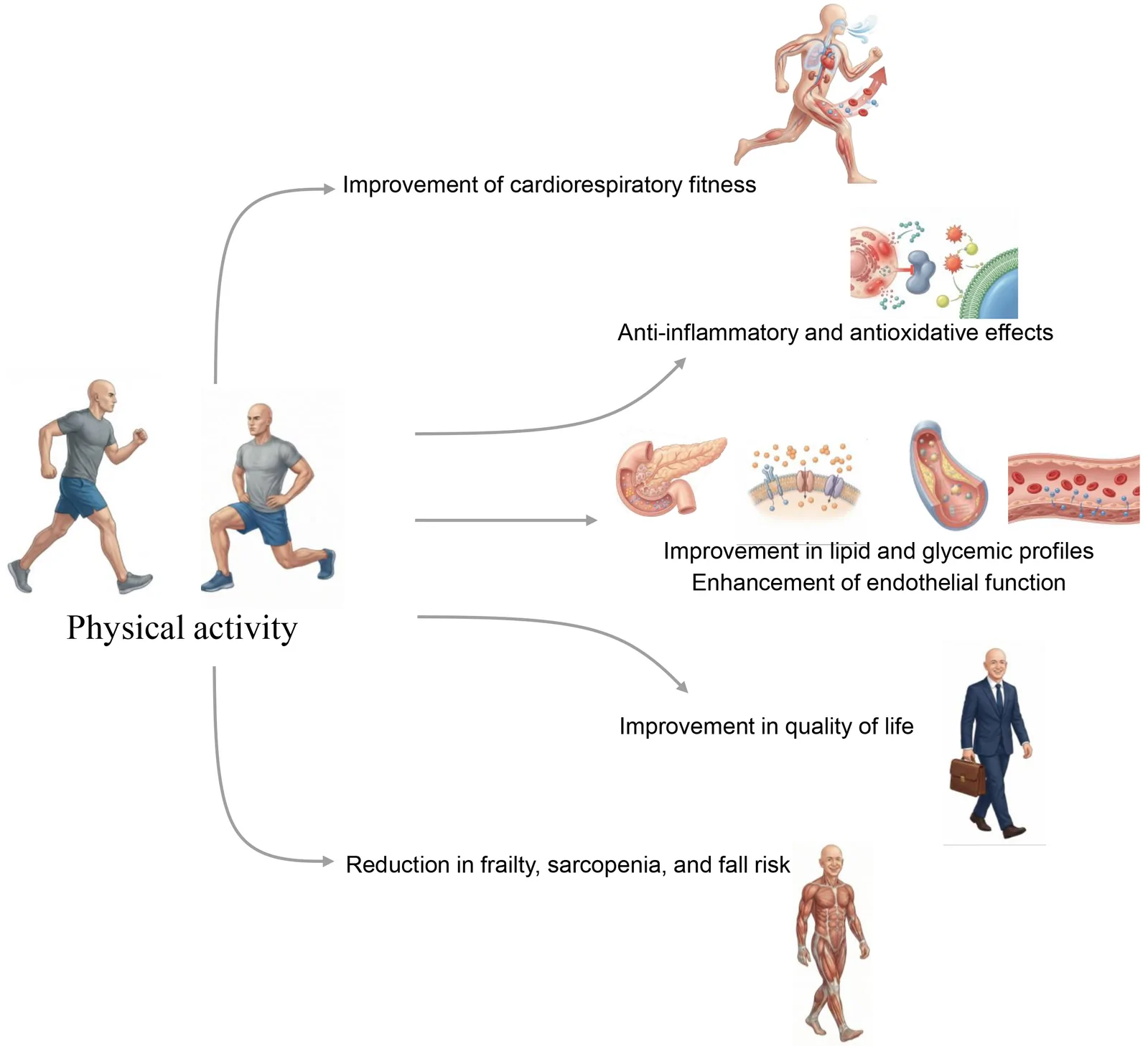
**Potential benefits of low-intensity physical activity in patients with arrhythmogenic cardiomyopathy**. In contrast to high-intensity or endurance exercise, carefully selected low-intensity physical activity may confer beneficial systemic and functional effects in patients with arrhythmogenic cardiomyopathy. These include improvements in cardiorespiratory fitness, endothelial function, lipid and glycemic profiles, and overall quality of life. Additional potential benefits comprise anti-inflammatory and antioxidative effects, preservation of musculoskeletal function, and reductions in frailty, sarcopenia, and fall risk. Such activity does not modify the underlying genetic disease substrate but may support long-term cardiovascular health and functional capacity when integrated into an individualized exercise prescription that avoids vigorous or competitive sports.

Although high-intensity and endurance exercise clearly accelerate disease progression and arrhythmic risk in ACM, complete physical inactivity is not a neutral alternative and may carry unintended physiological and psychosocial consequences (Fig. 1). Contemporary sports cardiology guidelines therefore distinguish between competitive or vigorous endurance sports and low-intensity physical activity, emphasizing individualized counselling that balances arrhythmic safety with preservation of overall health [9].

From a cardiovascular standpoint, light-to-moderate physical activity is associated with improvements in aerobic capacity, blood pressure regulation, insulin sensitivity, body composition, and inflammatory profile, factors that remain clinically relevant in patients with ACM, particularly with respect to long-term cardiometabolic health and comorbidity prevention [19]. Large epidemiological studies in general populations demonstrate a graded reduction in cardiovascular and all-cause mortality with increasing levels of moderate physical activity, arguing against a strategy of complete activity restriction in the absence of clear contraindications. Importantly, emerging ACM-specific data suggest that habitual lifestyle activity at non-athletic intensity does not confer the same arrhythmic burden observed with sustained endurance training [11,13]. In a multicenter study employing accelerometer-based activity assessment, low-intensity habitual physical activity was not associated with an increased incidence of rapid-rate non-sustained VT in patients with ACM [20]. Although these findings do not define a universally safe activity threshold, they support the distinction between everyday activity and high-intensity endurance exercise in terms of arrhythmic risk and are consistent with guideline recommendations permitting carefully selected recreational activity while discouraging competitive sport [9].

Beyond physiological considerations, allowing safe levels of physical activity may mitigate the psychosocial burden associated with ACM. Quality-of-life analyses indicate that younger age and ICD shocks are associated with impaired psychosocial outcomes, highlighting the importance of maintaining participation in daily life whenever possible [21]. Data from broader ICD populations further suggest that structured, supervised exercise does not necessarily increase ICD therapy burden, reinforcing the principle that appropriately prescribed, non-competitive activity can be compatible with device therapy, although disease-specific risk assessment remains essential [22]. In clinical practice, this supports a shared decision-making model incorporating phenotype, imaging findings, arrhythmic history, and genotype to guide individualized activity recommendations [9].

## 5. Desmosomal Dysfunction and Mechanical Stress

**Fig. 2.**
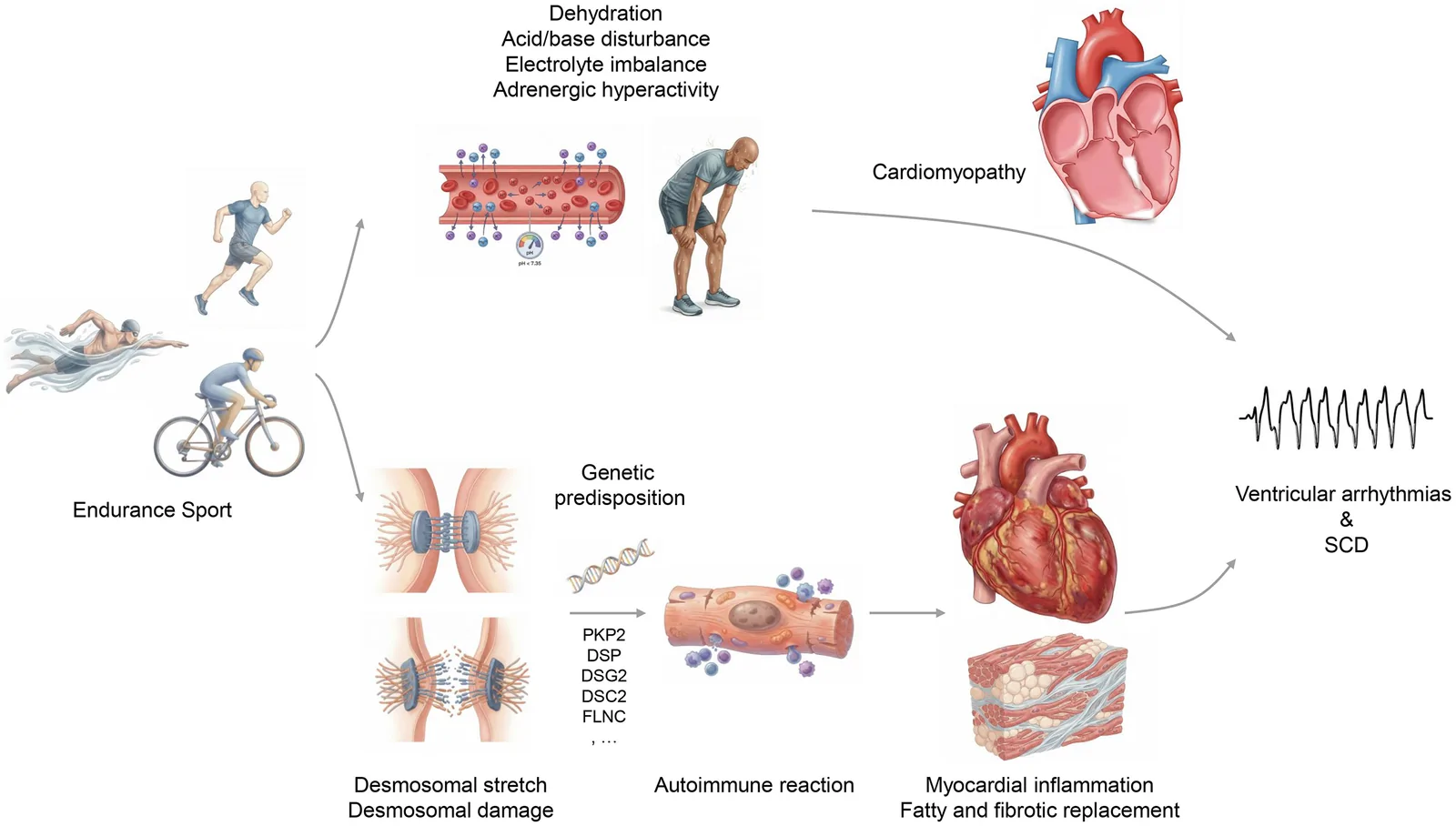
**Exercise-related mechanisms promoting disease progression and arrhythmogenesis in arrhythmogenic cardiomyopathy**. Endurance exercise imposes repetitive mechanical and hemodynamic stress on the myocardium, resulting in desmosomal stretch and dysfunction that promote cardiomyocyte detachment and cellular injury. In genetically predisposed individuals, pathogenic variants affecting desmosomal and cytoskeletal proteins create a vulnerable myocardial substrate that amplifies these effects. Exercise-related myocardial injury may be further exacerbated by inflammatory and autoimmune responses, leading to myocardial inflammation and progressive fibro-fatty replacement. In parallel, systemic exercise-related factors, including dehydration, electrolyte and acid–base disturbances, and adrenergic hyperactivity, act as dynamic modulators that lower arrhythmic thresholds. The interaction of structural remodelling and transient systemic stressors culminates in ventricular arrhythmias and sudden cardiac death, illustrating how endurance exercise may contribute to disease progression and arrhythmogenesis in ACM. *PKP2*, plakophilin-2; *DSP*, desmoplakin; *DSG2*, desmoglein-2; *DSC2*, desmocollin-2;* FLNC*, filamin C; SCD, sudden cardiac death; ACM, arrhythmogenic cardiomyopathy.

The majority of genetically explained classical ACM cases are caused by pathogenic variants in desmosomal genes, including plakophilin-2 (*PKP2*), desmoplakin (*DSP*), desmoglein-2 (*DSG2*), desmocollin-2 (*DSC2*), and junction plakoglobin (*JUP*) [2,23]. Overall, genetic testing identifies a pathogenic or likely pathogenic variant in approximately 50–60% of clinically diagnosed ACM cases, although the diagnostic yield varies according to cohort selection, phenotype, and sequencing strategy [24]. The proteins encoded by these genes are integral components of the intercalated disc, ensuring mechanical coupling between adjacent cardiomyocytes and maintaining structural integrity under conditions of repetitive mechanical load (Fig. 2) [25]. This anchoring function becomes particularly critical during sustained hemodynamic stress.

Endurance exercise induces prolonged increases in preload, wall stress, and cyclic myocardial stretch [9]. In the structurally normal heart, these stimuli lead to adaptive remodelling. In contrast, in ACM, compromised desmosomal integrity impairs the myocardium’s ability to withstand repetitive mechanical strain. Mechanical uncoupling promotes myocyte detachment, cellular injury, and apoptosis, ultimately resulting in progressive fibro-fatty replacement and ventricular remodelling [2,26]. Experimental models have consistently demonstrated that exercise exposure accelerates phenotypic expression, whereas exercise restriction delays structural progression and attenuates ventricular dysfunction [27,28]. Thus, desmosomal dysfunction provides a mechanistic basis for understanding why sustained endurance exercise may act as a disease modifier in genetically susceptible individuals.

## 6. Genotype-Specific Considerations in Exercise-Related ACM

### 6.1 *PKP2*-Associated ACM

Pathogenic variants in *PKP2*, encoding plakophilin-2, are among the most frequently identified genetic causes of ACM and have been most extensively studied in relation to exercise-related disease expression [17,28,29]. Plakophilin-2 is a key component of the desmosomal plaque at the intercalated disc and contributes to mechanical coupling, desmosomal assembly, and electrical stability through interactions with junctional and ion-channel complexes [28]. Reduced or dysfunctional plakophilin-2 expression may therefore lower myocardial tolerance to repetitive mechanical and adrenergic stress, particularly during sustained endurance exercise. Experimental and translational studies support a direct interaction between *PKP2* deficiency and exercise-related disease progression [28]. In plakophilin-2–deficient models, endurance exercise accelerates cardiac dysfunction, promotes arrhythmogenic remodelling of intracellular calcium handling, and increases susceptibility to ventricular arrhythmias [30]. These findings provide a mechanistic basis for clinical observations that cumulative exercise exposure is associated with earlier disease expression and higher arrhythmic burden in clinically ascertained carriers of desmosomal pathogenic or likely pathogenic variants [5]. Thus, *PKP2*-associated ACM illustrates how a desmosomal substrate may interact with exercise exposure to promote overt structural and arrhythmic disease [5,28]. However, this should not be interpreted as a uniform exercise prohibition for all *PKP2* variant carriers. Exercise recommendations should be individualized according to phenotype, ventricular involvement, arrhythmic history, family history, imaging findings, and the intensity and type of planned activity [1,9]. This is particularly relevant for genotype-positive, phenotype-negative relatives, in whom shared decision-making and longitudinal reassessment are essential to balance risk reduction against the adverse consequences of unnecessary inactivity.

### 6.2 *DSP*-Associated ACM and Inflammatory Myocardial Injury

Beyond this mechanically and electrically mediated disease model, inflammatory myocardial injury has emerged as an important additional mechanism in ACM, particularly in *DSP*-associated cardiomyopathy [31]. *DSP*-associated disease frequently presents with left-dominant or biventricular involvement and a distinct propensity for recurrent myocardial injury or myocarditis-like episodes [32]. An important clinical question is whether endurance exercise acts as a trigger, amplifier, or disease modifier in this inflammatory phenotype. Registry-based data indicate that endurance-level activity (>24 metabolic equivalent of task [MET]-hours/week) in carriers of pathogenic or likely pathogenic *DSP* variants is independently associated with myocardial injury episodes [33]. These observations suggest that exercise may promote disease progression not only through acute arrhythmic provocation, but also by facilitating inflammatory myocardial injury that may accelerate structural remodelling and scar formation (Fig. 2). Complementary multicenter analyses further demonstrate that myocarditis-like episodes in *DSP* cardiomyopathy are not benign findings, but are associated with adverse arrhythmic and heart-failure outcomes, particularly when recurrent [34]. Notably, initiation of immunosuppressive therapy during the first inflammatory episode was associated with improved outcomes in observational analyses, supporting the clinical relevance of inflammation in this context, although causality cannot be established from the available data [34].

From a mechanistic standpoint, prolonged high-intensity exercise induces transient immune activation and systemic inflammatory responses. Repetitive cycles of mechanical and hemodynamic stress may predispose to myocyte injury with secondary inflammatory amplification [35]. While exercise-related troponin release can occur in healthy endurance athletes, similar stressors in ACM, particularly in *DSP*-associated disease, act upon a structurally vulnerable myocardium and may result in disproportionate tissue injury and scar formation [35]. Consistent with this concept, inflammatory infiltrates and myocardial necrosis have been repeatedly documented in pathological series of ACM [36,37]. These mechanisms appear particularly relevant in left-dominant and biventricular ACM phenotypes, which frequently present with recurrent inflammatory episodes and are increasingly recognized in athletic populations [37]. Collectively, current evidence supports a multi-hit paradigm in which genetic susceptibility, sustained endurance exercise, mechanical stress, and inflammatory activation interact to promote recurrent myocardial injury, progressive fibrosis, and electrical instability.

## 7. Other Desmosomal and Non-Desmosomal Genes

Beyond *PKP2* and *DSP*, ACM may also be associated with pathogenic variants in other desmosomal genes, including *DSG2*, *DSC2*, and *JUP*, as well as non-desmosomal genes such as transmembrane protein 43 (*TMEM43*), phospholamban (*PLN*), desmin (*DES*), lamin A/C (*LMNA*), filamin C (*FLNC*), and others, depending on the diagnostic framework applied [1,38]. These genotypes differ substantially with respect to ventricular involvement, arrhythmic risk, heart-failure progression, and inflammatory or fibrotic phenotype [39]. For example, *PLN* and *FLNC* variants are often associated with left-dominant or dilated cardiomyopathy phenotypes and may carry a high arrhythmic risk, whereas *TMEM43*-associated disease may show particularly malignant arrhythmic expression in specific founder populations [40,41,42]. However, direct evidence defining genotype-specific exercise thresholds remains limited for most non-*PKP2* and non-*DSP* genotypes. Therefore, exercise counselling should avoid direct extrapolation from a single genetic subgroup to the entire ACM spectrum. Instead, recommendations should integrate the specific genotype with clinical phenotype, ventricular function, late gadolinium enhancement (LGE) or other evidence of myocardial scar, arrhythmic history, family history of SCD, and the patient’s intended exercise intensity. In this context, genotype should be considered as one component of a broader risk-stratification framework rather than as an isolated determinant of exercise eligibility.

From a clinical perspective, pathogenic or likely pathogenic variants may support diagnosis, enable cascade screening, and contribute to genotype-informed follow-up and risk stratification [43]. In contrast, variants of uncertain significance (VUS) should not be considered disease-causing and should not be used as the sole basis for diagnosis, family screening, exercise restriction, or therapeutic decisions; instead, they require periodic reinterpretation in the context of phenotype, segregation data, and updated variant databases [43].

Genotype should be interpreted in conjunction with the clinical phenotype, because right-dominant, left-dominant, and biventricular ACM differ in disease expression, progression, and arrhythmic risk [38]. In classical desmosomal ACM, *PKP2* variants are most commonly associated with a right-dominant phenotype, whereas *DSP* variants frequently show left-dominant or biventricular involvement and may present with myocarditis-like episodes and myocardial scar [44]. In addition, genes such as *FLNC*, *LMNA*, and *PLN* are increasingly recognized in arrhythmogenic left ventricular or arrhythmogenic-dilated phenotypes and may be associated with a relevant ventricular arrhythmia risk [1,38]. Therefore, genotype and phenotype should be used to refine individualized risk assessment in exercise counselling, but should not define exercise eligibility in isolation.

## 8. Arrhythmogenesis Beyond the Exercise Period

Vigorous physical exertion has been implicated in the acute initiation of ventricular tachyarrhythmias, primarily through adrenergic activation and transient hemodynamic stress [37]. However, in the context of ACM, the risk of arrhythmia extends beyond the exercise period. Longitudinal cohort studies consistently demonstrate that cumulative exercise exposure accelerates the structural progression of the disease, fostering the development of a persistent arrhythmogenic substrate [5,7,15]. This substrate, characterized by fibrotic replacement, ventricular dilation, and electrical instability, predisposes patients to arrhythmias not only during exercise but also at rest, in the early recovery phase, and during routine daily activities. Furthermore, these structural alterations often persist even after the cessation of competitive sport, underscoring the role of exercise not only as an acute trigger but also as a long-term modifier of disease trajectory [5].

Evidence from ICD cohorts further supports this substrate-driven risk. In the prospective multinational ICD Sports Registry, appropriate ICD therapies occurred not only during sports participation but also in non-sport settings, underscoring that malignant ventricular arrhythmias frequently arise outside structured training or competition [16]. In ACM populations specifically, reduction of exercise intensity following diagnosis is associated with a lower incidence of arrhythmic events; however, events are not abolished, particularly in patients with established structural remodelling [11,13,45]. These observations are consistent with the concept that once fibrotic and electrical substrate has developed, arrhythmic risk becomes only partially reversible. From a clinical perspective, discontinuation of competitive endurance sport represents an important risk-reduction strategy but does not eliminate arrhythmic susceptibility. Ongoing surveillance, individualized risk stratification, and adherence to guideline-directed management remain essential even after exercise restriction, particularly in patients with documented structural disease or prior ventricular arrhythmias [18].

## 9. Systemic and Exercise-Related Modulators of Arrhythmic Risk

Beyond structural remodelling intrinsic to ACM, physical exercise may transiently enhance arrhythmic susceptibility through systemic mechanisms that are not disease-specific but may exert amplified effects in a vulnerable myocardium. Prolonged or high-intensity exertion is frequently associated with electrolyte disturbances, including hypokalemia, hypomagnesemia, and exercise-associated hyponatremia, all of which are recognized facilitators of ventricular arrhythmogenesis (Fig. 2) [46,47]. Even modest alterations in serum electrolyte concentrations can influence myocardial repolarization and conduction velocity, thereby lowering arrhythmic thresholds in individuals with underlying structural or electrical instability. Endurance exercise is also characterized by substantial autonomic fluctuations. Sustained sympathetic activation during exertion is followed by a rapid shift toward parasympathetic predominance during early recovery. Both autonomic states have been implicated in the initiation of ventricular arrhythmias, particularly in the presence of pre-existing arrhythmogenic substrate. Long-term rhythm monitoring data in endurance athletes indicate that ventricular arrhythmias frequently occur during recovery or at rest rather than at peak exertion, underscoring the importance of autonomic imbalance and substrate vulnerability as critical determinants of arrhythmic events [48].

Additional contributors include exercise-induced dehydration, intravascular volume redistribution, and transient alterations in myocardial repolarization, each of which may destabilize electrical homeostasis [35,49,50]. Although these factors are not unique to ACM, their electrophysiological consequences are likely magnified in the presence of desmosomal dysfunction, fibrotic remodelling, or ventricular dilation. Collectively, these findings support a model in which exercise-related arrhythmic risk reflects the interaction between a fixed structural substrate and dynamic systemic stressors, helping to explain the occurrence of malignant arrhythmias outside periods of active exertion.

## 10. Conclusion

Physical activity represents a major determinant of disease expression in ACM. Robust clinical and experimental evidence demonstrates that sustained high-intensity and endurance exercise accelerates structural remodelling, promotes electrical instability, and increases the incidence of malignant ventricular arrhythmias. Importantly, the adverse consequences of cumulative exercise exposure extend beyond periods of exertion, as progressive fibrosis and substrate formation confer persistent arrhythmic vulnerability even after discontinuation of competitive sport. At the same time, current data support a differentiated approach to physical activity. While vigorous and competitive endurance sports should be avoided, low-intensity, non-extensive lifestyle activity appears to impose substantially less arrhythmic burden and may contribute to the preservation of functional capacity, cardiometabolic health, and psychosocial well-being. Management therefore requires individualized counselling that integrates genotype, phenotype, imaging findings, and arrhythmic history. Future prospective studies are needed to better define intensity thresholds, clarify genotype-specific responses, and develop evidence-based exercise prescriptions that reconcile arrhythmic risk reduction with long-term health preservation in patients with ACM.
